# Perinatal Outcomes after Liver Transplantation: Is There a Role for Aspirin Treatment?

**DOI:** 10.3390/jcm12113733

**Published:** 2023-05-29

**Authors:** Gil Zeevi, Marius Braun, Eviatar Nesher, Arnon Wiznitzer, Asnat Walfisch, Eran Hadar, Alyssa Hochberg

**Affiliations:** 1 Helen Schneider Hospital for Women, Rabin Medical Center, 39 Jabotinsky St., Petach Tikva 4941492, Israel; 2 Sackler Faculty of Medicine, Tel Aviv University, Ramat Aviv, P.O. Box 39040, Tel Aviv 6997801, Israel; 3 Liver Institute, Rabin Medical Center, 39 Jabotinsky St., Petach Tikva 4941492, Israel; 4 Department of Organ Transplantation, Rabin Medical Center, 39 Jabotinsky St., Petach Tikva 4941492, Israel

**Keywords:** liver transplantation, aspirin, pre-eclampsia, perinatal outcomes, prevention

## Abstract

Background: We aimed to describe perinatal outcomes and evaluate aspirin treatment effects in liver-transplanted pregnant women. Methods: A retrospective study examining perinatal outcomes in liver transplant recipients at a single center (2016–2022). The effect of low-dose aspirin treatment on the risk of developing hypertensive disease in these patients was evaluated. Results: Fourteen deliveries in 11 pregnant liver transplant recipients were identified. Primary liver disease was Wilson’s in 50% of pregnancies. The median age was 23 years at transplant and 30 at conception. Tacrolimus was administered in all, steroids in 10 (71.43%), and aspirin (100 mg daily) in 7 (50.0%). Overall, two women (14.28%) developed preeclampsia, and one (7.14%) developed gestational hypertension. Median gestational age at delivery was 37 weeks (31–39 weeks), with six preterm births (between 31–36 weeks) and a median birthweight of 3004 g(range 1450–4100 g). None of those receiving aspirin developed hypertensive disease or suffered excessive bleeding during pregnancy, compared to two (28.57%) with pre-eclampsia in the non-aspirin group. Conclusion: Liver-transplanted pregnant women comprise a unique and complex patient population with overall favorable pregnancy outcomes. Based on our single-center experience and due to its safety profile and potential benefit, we recommend low-dose aspirin in all liver transplanted patients during pregnancy for preeclampsia prevention. Further large prospective studies are needed to corroborate our findings.

## 1. Introduction

During the past 10 years, an increasing number of pregnancies achieved following liver transplantation has been observed. These pregnancies are at increased risk for both maternal and neonatal complications [1]. Maternal complications include hypertensive disorders of pregnancy, gestational diabetes, post-partum hemorrhage, blood product transfusions, graft rejection, and a higher rate of cesarean deliveries [2]. Neonatal complications include preterm birth, intrauterine growth restriction, and fetal distress, with no known increase in the incidence of congenital anomalies [1,3].

Since the report of the first case of a pregnancy following a liver transplant over 40 years ago [4], major progress has been made in the fields of transplantation and obstetrics. This may be attributed to new surgical techniques, better anesthetic care, and improved immunologic and pharmacological treatments. Yet, there is no consensus regarding optimal treatment and surveillance during pregnancy for this unique population. The obstetric field has also undergone great progress in the prevention of pre-eclampsia in high-risk patients, including kidney transplant recipients, through early treatment with low-dose aspirin between 12–16 weeks of gestation [5,6,7,8]. A recent systematic review summarized the key clinical guidelines on aspirin use during pregnancy for preventing pre-eclampsia and fetal growth restriction. The authors cite the major indications for aspirin intake during pregnancy, including previous preeclampsia and maternal medical co-morbidity, such as chronic hypertension, autoimmune disease, and diabetes mellitus. Among the various guidelines, there remains variability regarding the recommended aspirin dosage and gestational age upon treatment commencement and cessation [5]. However, most guidelines recommend early treatment with low-dose aspirin (75–150 mg) between 12–16 weeks of gestation [6,7,8,9].

Our tertiary university-affiliated medical center is the largest solid organ transplant center in Israel, centralizing the care of more than 300 transplant recipients each year, with the performance of 70 liver transplants annually. When pregnant, obstetric follow-up is performed by a multidisciplinary team comprised of a Maternal-Fetal-Medicine specialist, a hepatologist, and a transplant surgeon. In our study, the first case series from Israel, we present our single-center experience managing pregnancies following liver transplantation during the past 6 years and describe maternal, fetal, neonatal, and allograft outcomes. In the present study, we also examined the effect of routine low-dose aspirin use during pregnancy on hypertensive disease incidence, a policy that has been implemented in pregnant liver-transplant recipients at our institution since 2018.

## 2. Materials and Methods

We performed an observational retrospective study examining 14 deliveries among 11 liver-transplant recipients delivered between 2016 and 2022 at our tertiary medical center. Complete data for retrospective analysis were retrieved from the comprehensive computerized hepatology clinic and perinatal database of our center and were cross-tabulated using an individualized identification number per patient. Data from the neonatal unit and the neonatal intensive care unit (NICU) admissions were integrated into the delivery room database using the unique admission number assigned to each woman and her offspring.

Included in the study were all pregnant liver transplant recipients managed at our medical center during the study period. Of note, all had complete perinatal data available for analysis. 

The following maternal parameters before and during pregnancy were assessed: primary liver disease, age at liver transplantation, age at conception, the time interval between transplantation and conception, immunosuppressive regimen received, pre-pregnancy co-morbidities (diabetes mellitus and hypertension), gravidity, parity, and aspirin use.

The obstetric characteristics and maternal outcomes evaluated included: induction of labor, mode of delivery, preterm birth (<37 weeks of gestation), gestational hypertension, pre-eclampsia, gestational diabetes mellitus, cholestasis, anemia, urinary tract infections and other infections, placental abruption, graft rejection, postpartum fever, postpartum hemorrhage, and blood transfusion.

Neonatal outcomes were assessed and included: gestational age at delivery; birthweight; Apgar score, umbilical cord pH; neonatal death; jaundice requiring phototherapy; NICU admission; adverse respiratory composite outcome (including respiratory distress syndrome (RDS), transient tachypnea of the newborn (TTN) or need for mechanical ventilation); and sepsis.

### Statistical Analysis

Data were analyzed using descriptive statistics, as this was an observational study consisting of a small sample size. Continuous variables are presented as median (range), and categorical variables as numbers (percentages).

The study was approved by the Rabin Medical Center Review Board (RMC 0339-19). Informed consent was waived due to the retrospective design of the study.

## 3. Results

We identified 14 deliveries in 11 pregnant liver transplant recipients (Table 1). The indications for liver transplantation included: acute liver failure not otherwise specified (3 recipients), Wilson’s disease (5 recipients, all with liver cirrhosis), liver failure caused by Hepatitis A virus (1 recipient), primary sclerosing cholangitis (1 recipient), and hyperoxaluria type 1 (1 recipient).

Three patients suffered from portal hypertension prior to transplantation. The median age at liver transplant was 23 (range 2–33), the median age at conception was 30 (range 25–35), and the median time between organ transplantation and conception was 4.5 years (range 0–26 years). Six women were nulliparous, two had pre-gestational diabetes and one suffered from depression (Table 2). All recipients were treated with tacrolimus in all pregnancies, and steroids were administered to 10 (71.43%). Low-dose aspirin (100 mg daily) was administered at our institution to pregnant liver-transplant recipients beginning in 2018 and was given in seven (50%) of the pregnancies described here (Table 2).

### 3.1. Maternal and Neonatal Outcomes

Table 3 presents maternal outcomes: two women (14.28%) suffered from pre-eclampsia, four (28.57%) from diabetes of any kind (pre-gestational diabetes mellitus or gestational diabetes), and six (42.86%) delivered by cesarean section for various indications. The indications for cesarean sections included: three due to abnormal fetal presentation (two with breech and one with compound presentation), one due to a non-reactive fetal monitor with repeated variable decelerations and an abnormal biophysical profile, one due to liver transplantation during pregnancy, and one due to previous CS. There were no cases of maternal death. Elevated liver enzymes (aspartate transaminase (AST) and alanine transaminase (ALT) elevations only, with no rise in bilirubin levels) were observed in 6 patients and resolved postpartum, with no cases of acute graft rejection identified. Cholestasis occurred in three cases (21.43%), thrombocytopenia in 5 (35.71%), and infection (UTI, bacteremia, sepsis, endometritis) in four (28.57%). Post-partum hemorrhage occurred in two cases (14.28%), with one woman requiring blood products transfusion. There were no cases of placental abruption or admissions to the intensive care unit during pregnancy or following delivery. 

Neonatal outcomes are presented in Table 4: median gestational age at delivery was 37 weeks (range 31–39 weeks), with six (42.86%) women experiencing preterm birth (range 31–36 weeks), with a median birthweight of 3004 g (range, 1450–4100 g). Five-minute Apgar scores were all >8, and umbilical cord pH was >7.15 in all cases; however, two neonates (14.28%) were admitted to the neonatal intensive care unit. No cases of sepsis, asphyxia, intrauterine fetal death, or neonatal death were observed. 

The characteristics and obstetric and neonatal outcomes of pregnancies stratified by aspirin treatment are presented in Table 5. None of the seven women receiving low-dose aspirin (beginning at 12 weeks of gestation and continuing until 36 weeks) developed hypertensive disease or preeclampsia, while two of the seven women not receiving aspirin (28.57%) developed mild preeclampsia, and one (14.28%) developed gestational hypertension. Of note, in the non-aspirin group one, of the women who developed mild preeclampsia had diabetes mellitus, and one was also a kidney transplant recipient (due to Hyperoxaluria type 1). Another patient in the non-aspirin group who had diabetes mellitus did not go on to develop preeclampsia, and the single patient who developed gestational hypertension did not have any risk factors for preeclampsia. None of the aspirin-treated patients suffered excessive bleeding during pregnancy. 

The non-aspirin group, as compared to the aspirin-treated group, had higher rates of overweight (BMI > 25 kg/m^2^), 28.57% vs. 14.28%, respectively), diabetes mellitus (28.57% vs. 0%), kidney disease (14.28% vs. 0%), and nulliparity (57.1% vs. 28.57%). None of the women in either group suffered from chronic hypertension or had a history of preeclampsia in previous pregnancies. A higher percentage of women in the aspirin group were treated with prednisone, as compared to the non-aspirin group (85.7% versus 57.1%, respectively). Women in the aspirin group had higher rates of gestational diabetes (28.57% vs. 0%) and post-partum hemorrhage due to uterine atony (28.57% vs. 0%). There were no cases complicated by placental abruption or fetal growth restriction in the entire cohort. 

At our institution, pregnant liver transplant recipients do not undergo routine Doppler flow assessment in the uterine arteries in the first or second trimesters unless there is an indication to do so (such as a history of chronic hypertension, previous preeclampsia, etc.). Only one patient, who underwent a liver transplant subsequent to Wilson’s disease, with no other risk factors for preeclampsia, had a routine measurement of the pulsatility index in the uterine arteries on her first fetal anatomy scan (week 15) for no specific indication, which was found to be normal. Additionally, doppler flows are only performed later in gestation at our center in cases of fetal growth restriction. However, as noted, no woman in our cohort had fetal growth restriction; hence, Doppler flows were not performed. 

### 3.2. Two Unusual Pregnancies

One woman in our cohort underwent liver transplantation in her 23rd week of gestation due to acute liver failure attributed to propylthiouracil (PTU) use. She delivered by cesarean section at 31 weeks due to elevated liver enzymes (AST, ALT, bilirubin, and Gamma-glutamyl Transferase (GGT)).

Another patient, a liver transplant recipient subsequent to Wilson’s disease with pre-gestational diabetes and depression, presented at 32 weeks of gestation with sepsis (caused by extended-spectrum beta-lactamase (ESBL) positive Escherichia coli and Klebsiella pneumoniae) and fever and was delivered by cesarean section due to a non-reactive fetal monitor with repeated variable decelerations and an abnormal biophysical profile. Placental and uterine cultures were all found to be negative.

## 4. Discussion

In this summary of our single-center institutional experience, we demonstrated that pregnancy after liver transplantation holds favorable results for the mother, neonate, and allograft. No maternal or fetal deaths occurred, and there was no acute allograft rejection. 

Despite the limited number of cases described in the literature, our results match larger case series outcomes, such as that depicted by Kubo et al. [3], with the incidence of preterm birth being 48%, cesarean section rate 46%, pre-eclampsia 16%, and low birth weight 24%. A recent study examining perinatal outcomes in 14 liver transplant recipient pregnancies also found similar perinatal outcomes in these patients, with a general gestational age at delivery of 36.67 weeks (range 31–40); 41.67% preterm birth rate (range 31–36 weeks); mean birthweight of 2775.83 g (range 1140–3600 g); and an Apgar score>8 in almost all neonates [10]. However, they demonstrated a slightly higher cesarean delivery rate of 58.33% compared to that in our cohort of gestations (42.86%). Additionally, none of the women in their cohort received aspirin treatment. Regarding the immunosuppressive medication regimen in our cohort, it is in accordance with the treatment recommended in such cases in previous publications, with all patients receiving tacrolimus and most receiving steroids [1,2,10]. 

Low-dose aspirin treatment for pregnant liver transplant recipients, with the aim of reducing the risk for preterm preeclampsia [5], began in 2018 at our institution after the results of the ASPRE study [7] and owing to the subsequent favorable results and effects we saw at our institution with such treatment in pregnant kidney transplant recipients [6,7,9]. As noted, pregnant liver transplant recipients at our center began receiving routine aspirin treatment at 12–16 weeks of gestation as part of a policy change in 2018, regardless of other risk factors for preeclampsia. Treatment was discontinued at 36 weeks of gestation or at delivery if it occurred earlier. 

The incidence of pre-eclampsia in pregnant liver transplant recipients in our study was 14.28%. No case of pre-eclampsia was seen in the aspirin-treated group, compared to a rate of 28.57% in the non-aspirin group (Table 5). We evaluated risk factors for preeclampsia development in our cohort of patients and found that women who did not receive aspirin had higher rates of increased BMI (>25 kg/m^2^), diabetes mellitus, kidney disease, and nulliparity compared to women in the aspirin-treated group. However, more women in the aspirin-treated group received prednisone during gestation, suffered from gestational diabetes, and experienced post-partum hemorrhage due to uterine atony. No woman in either group suffered from chronic hypertension or had a history of preeclampsia in previous pregnancies. Notably, the two women in the non-aspirin group who went on to develop preeclampsia actually had an indication for aspirin treatment (a patient with a kidney transplant and a patient with diabetes mellitus) but did not receive it. Another patient with diabetes mellitus in the non-aspirin group did not develop preeclampsia despite not receiving aspirin treatment. 

Our findings are challenging to interpret in the context of an observational study with a limited sample size, making it difficult to ascertain whether risk factors for preeclampsia development present in the non-aspirin group contributed to preeclampsia development in these patients. However, one cannot ignore the favorable results and absence of preeclampsia in the aspirin-treated group. 

Regarding the increased rate of post-partum hemorrhage in the aspirin-treated group, we could not postulate a connection between aspirin treatment and uterine atony in these cases (which was documented as the reason for post-partum hemorrhage). Furthermore, in both cases complicated by post-partum hemorrhage in the aspirin group, aspirin treatment was stopped more than one week prior to delivery. 

Whilst previous studies have reported rates ranging between 21–26% for pre-eclampsia in liver-transplanted pregnant women, more recent studies reported a lower incidence for pre-eclampsia ranging between 7–12% [1,3,8]. Additionally, a recent retrospective study by Nachshon et al. [11] examined the incidence of pre-eclampsia in 311 pregnant women with pre-existing chronic liver disease (including viral and autoimmune hepatitis, non-alcoholic fatty liver, Wilson disease, and cirrhosis) compared to 933 healthy pregnant controls. They showed that pre-existing chronic liver disease was associated with a 2.6-fold increased risk of pre-eclampsia. In this study, pre-eclampsia was diagnosed in 28 women (9.0%) in the study group and 33 women (3.54%) in the control group (*p* < 0.001). On multivariate analysis, chronic liver disease was found to be an independent risk factor for pre-eclampsia (aOR 2.631, 95% CI 1.518–4.561), though this study did not specify the inclusion or exclusion of liver transplant recipients. 

Preeclampsia, a form of hypertension unique to human pregnancy, is considered a complex, multisystem heterogeneous condition that may originate from multiple causes. It is believed to evolve from changes in placental development that result in placental ischemia, thereby producing inflammation and oxidative stress. This stress may be a result of pathological placental development and placental ischemia, an overactive inflammatory response to normal placentation, or a pre-existing inflammatory condition. Organ transplant recipients hold at least two of these risk factors–a higher risk for pathological placental development and a pre-existing inflammatory state [6]. In liver-transplanted pregnant patients, it is also possible that the underlying chronic liver disease that led to liver transplantation in itself increases the risk for pre-eclampsia. The use of drugs with anti-inflammatory, anti-angiogenic, and antiplatelet properties, such as low-dose aspirin, holds a preventive effect on the risk for preeclampsia development. A recent review by Rahim et al. [8] linked this preventive effect to increased management of immunosuppression and risk factors associated with pre-eclampsia. Rahim’s team also recommended daily aspirin treatment starting before 16 weeks of gestation in order to improve placental hemodynamics and reduce the risk for preterm pre-eclampsia in women at risk. Though based on retrospective data and a limited number of patients, we cautiously postulate that aspirin treatment may have a preventive effect on preeclampsia development in pregnant liver-transplanted women and feel this is an area to be explored in large, prospective, multicenter studies. 

Liver-transplanted women are also at increased risk for other pregnancy complications, such as postpartum hemorrhage and the need for blood transfusions. Hematologic findings are postulated to be related to both chronic immunosuppressive therapy and physiological changes of pregnancy leading to anemia [12,13]. In our cohort, two women (14.28%) suffered postpartum hemorrhage, with one (7.14%) requiring a blood transfusion. One parturient with postpartum hemorrhage had known low platelets secondary to long-lasting splenomegaly, and the other had a normal platelet count without any known clotting factor deficiencies. Of note, all women in our cohort had a functional liver without cirrhosis, and no woman receiving low-dose aspirin suffered excessive bleeding during pregnancy.

Post-partum infection in liver transplant recipients is another pregnancy-related complication obstetricians need to be aware of. In our study, three women were diagnosed with sepsis due to a urinary tract infection (UTI), and another patient was diagnosed with endometritis. This is higher than the rate in the general obstetric population, where 3% suffer from UTI post-partum and 2% from endometritis [14], most probably secondary to immunosuppression in this patient population. Expeditious diagnosis and treatment may be life-saving in these immunosuppressed women, and therefore signs of infection should be sought after and treated as soon as they appear.

The strength of our study lies in the fact that it was performed at a single large tertiary center that centralizes and specializes in the care of these complex patients, with standardized hepatic and obstetrical follow-up and management. Additionally, to the best of our knowledge, this is the largest series to date reporting on the outcomes of treatment of these women with low-dose aspirin with the aim of preeclampsia prevention. 

Our study has several limitations. First, the small number of cases included precludes reaching statistically significant conclusions regarding outcomes and management in this patient population. Having said that, due to the rarity of the entity of pregnant liver-transplanted women, reaching a proper sample size for adequate power is challenging, especially at a single center. We believe future studies combining multi-center experiences may shed light on this important and complex group of pregnant women. Another limitation lies in the observational and retrospective nature of our study, requiring future validation in prospective studies, especially with regard to low-dose aspirin treatment in this patient population. 

## 5. Conclusions

Liver-transplanted pregnant women comprise a unique and complex patient population due to their underlying chronic medical condition, immunosuppressive drug regimen, and increased risk for pre-eclampsia, thus necessitating close surveillance by a multidisciplinary team. The pregnancy outcomes are generally favorable. Based on our single-center experience, we cautiously conclude that low-dose aspirin treatment beginning at 12–16 weeks of gestation should be routinely prescribed for liver-transplanted pregnant women, with the aim of preventing pre-eclampsia and its associated sequelae. We feel that given the proven safety of this intervention coupled with the suggested benefit-this recommendation is justifiable. There is a need for further, large, multi-center prospective studies on this subject to validate our findings.

## Figures and Tables

**Table 1:** Primary Liver Diseases.

Primary Liver Disease	Number of Women n = 11	Number of Pregnancies n = 14
Wilson	5 (45.45)	7 (50)
Acute liver failure NOS ^a^	3 (27.27)	3 (21.43)
HAV ^b^	1 (9.09)	2 (14.28)
Hyperoxaluria type 1	1 (9.09)	1 (7.14)
PSC ^c^	1 (9.09)	1 (7.14)

Continuous variables are presented as median (range) and categorical variables as n (%). ^a^ NOS = Not otherwise specified; ^b^ HAV = Hepatitis A virus; ^c^ PSC = Primary sclerosing cholangitis.

**Table 2:** Characteristics of the 14 pregnancies.

Age at liver transplant (years)	23 (2–33)
Age at conception (years)	30 (25–35)
**Transplant-to-conception interval**	
Less than 2 years	2 (1 during pregnancy)
More than 2 years	12
Gravidity	2 (1–5)
**Parity**	
0	6 (42.86)
1	5 (35.71)
2	3 (21.43)
Diabetes Mellitus	2 (14.28)
Hypertension	0 (0)
**Body mass index (kg/m^2^)**	
Normal (18–25)	11 (78.57)
>25	3 (21.43)
**Immunosuppressive Medications**	
Tacrolimus (Prograf)	14 (100)
Prednisone	10 (71.43)
Aspirin	7 (50.0)

Continuous variables are presented as median (range) and categorical variables as n (%).

**Table 3:** Obstetric characteristics and maternal outcomes of the 14 pregnancies.

Variable	Pregnancies N (%)
Induction of labor	5 (35.71)
**Mode of delivery**	
Normal vaginal delivery	7 (50)
Vacuum extraction delivery	1 (7.14)
Cesarean section	6 (42.86)
Preterm birth (<37 weeks)	6 (42.86)
Gestational hypertension	1 (7.14)
Preeclampsia	2 (14.28)
Gestational diabetes	2 (14.28)
Thrombocytopenia	5 (35.71)
Cholestasis	3 (21.43)
Elevated liver enzymes	6 (42.86)
Postpartum infection	4 (28.57)
Postpartum hemorrhage	2 (14.28)
Severe anemia	1 (7.14)
Blood transfusion	1 (7.14)
Graft rejection	0 (0)

**Table 4:** Neonatal outcomes in the 14 pregnancies.

Neonatal Outcome	Pregnancies N (%)
**5-min Apgar score**	
Less than 7	0 (0)
More than 7	14 (100)
**Gestational age at delivery**	
Less than 32 weeks	1 (7.14)
32–36 + 6 weeks	5 (35.71)
≥37 weeks	8 (57.14)
**Birthweight**	
1000–1500 g	1 (7.143)
1500–2500 g	2 (14.28)
>2500 g	11 (78.57)
Congenital anomalies	0 (0)
Neonatal intensive care unit (NICU) admission	2 (14.28)
PH < 7.15	0 (0)
Neonatal death	0 (0)
Jaundice requiring phototherapy	1 (7.14)
Respiratory composite ^a^	1 (7.14)
Blood transfusion	1 (7.14)
Sepsis	0 (0)

Continuous variables are presented as median (range) and categorical variables as n (%). ^a^ Respiratory composite = respiratory distress syndrome (RDS), transient tachypnea of the newborn (TTN), or need for mechanical ventilation.

**Table 5:** Characteristics and obstetric and neonatal outcomes of pregnancies stratified by aspirin treatment.

	Aspirin n = 7 (%)	No Aspirin n = 7 (%)
**Maternal characteristics**		
Age at delivery (years)	31 (29–35)	30 (26–34)
BMI ^a^ >25 kg/m^2^	1(14.3)	2 (28.6)
Diabetes mellitus	0 (0)	2 (28.6)
Chronic hypertension	0 (0)	0 (0)
Kidney disease ^b^	0 (0)	1(14.3)
History of preeclampsia in previous pregnancies	0 (0)	0 (0)
Prednisone treatment in current pregnancy	6 (85.7)	4 (57.1)
Prednisone dose (mg)	7.5 (2.5–20)	12.5 (5–30)
Nulliparity	2 (28.6)	4 (57.1)
**Pregnancy and delivery outcomes**		
Gestational diabetes	2 (28.6)	0 (0)
Gestational hypertension	0 (0)	1(14.2)
Preeclampsia	0 (0)	2 (28.4)
Fetal growth restriction	0 (0)	0 (0)
Placental abruption	0 (0)	0 (0)
Preterm birth (<37 weeks)	3 (42.9)	2 (28.6)
Normal vaginal delivery	4 (57.1)	3 (42.86)
Vacuum extraction delivery	1(14.3)	0 (0)
Cesarean section	2 (28.6)	4 (57.1)
Postpartum hemorrhage	2 (28.6)	0 (0)
Blood transfusion	1(14.3)	0
**Neonatal outcomes**		
Low birthweight (<2500 g)	1(14.3%)	2 (28.6)
NICU ^c^	0 (0)	2 (28.6)

Continuous variables are presented as median (range) and categorical variables as n (%).^a^ BMI = body mass index; ^b^ Kidney disease = kidney transplant recipient due to Hyperoxaluria type 1; ^c^ NICU = neonatal intensive care unit.

## Data Availability

Not applicable.
